# Histone H2B ubiquitination mediated chromatin relaxation is essential for the induction of somatic cell reprogramming

**DOI:** 10.1111/cpr.13080

**Published:** 2021-06-22

**Authors:** Liying Wang, Zhiliang Xu, Libin Wang, Chao Liu, Huafang Wei, Ruidan Zhang, Yinghong Chen, Lina Wang, Wenwen Liu, Sai Xiao, Wei Li, Wei Li

**Affiliations:** ^1^ State Key Laboratory of Stem Cell and Reproductive Biology Institute of Zoology Chinese Academy of Sciences Beijing China; ^2^ University of the Chinese Academy of Sciences Beijing China; ^3^ Department of Obstetrics and Gynecology Key Laboratory for Major Obstetric Diseases of Guangdong Province Key Laboratory of Reproduction and Genetics of Guangdong Higher Education Institutes The Third Affiliated Hospital of Guangzhou Medical University Guangzhou China

**Keywords:** cell reprogramming, chromatin relaxation, histone H2B ubiquitination, histone modification

## Abstract

**Objectives:**

Cell reprogramming has significant impacts on their potential application in regenerative medicine. Chromatin remodelling plays a very important role in cell reprogramming, but its underlying mechanism remains poorly understood.

**Materials and methods:**

RNA‐seq, quantitative RT‐PCR and western blot analysis were applied to study the role of RNF20 and H2B ubiquitination during mouse somatic cell reprogramming. Chromatin structure and the recruitment of transcription factors were analysed by ChIP‐seq, micrococcal nuclease sensitivity assays and immunofluorescence staining.

**Results:**

We show that RNF20 is highly expressed at the early stage of reprogramming along with the accumulation of H2B ubiquitination at the same stage, and *Rnf20* knockout results in the failure of reprogramming at the initial stage but not the maturation and stabilization stages. RNA‐seq showed that *Rnf20* knockout mainly affects the early stage of cell reprogramming by impairing the transcription of MET‐related genes and early pluripotency genes. Importantly, *Rnf20* knockout results in a more compacted chromosomes structure in reprogrammable cells, suppressing the recruitment of reprogramming transcription factors to their proper locations on the chromosomes, and finally resulting in the failure of pluripotent gene network establishment.

**Conclusions:**

Histone H2B ubiquitination mediated chromatin relaxation is essential for the induction of somatic cell reprogramming.

## Graphical abstract

RNF20 and H2Bub are highly expressed in the early stages of cell reprogramming, and *Rnf20* knockout mainly affects the early stages of cell reprogramming. The deficiency of RNF20 in reprogrammable cells affected chromatin relaxation by reducing H2B ubiquitination, thus preventing the recruitment of SOX2 to the proper positions on the chromosome and repressing the transcription of pluripotency genes and MET‐related genes during the early stages of cell reprogramming.

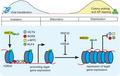

## 1 INTRODUCTION

Somatic cell reprogramming is essential to regenerative medicine, and reprogramming of somatic cells into induced pluripotent stem cells (iPSCs) can be achieved by the expression of only four transcription factors (TFs), OCT4, SOX2, KLF4 and c‐MYC (collectively termed OSKM).^1^ This process can be divided into three phases termed initiation, maturation and stabilization.^2^ At the early phase, many physiological events such as RNA processing, cell cycle, DNA repair and mesenchymal‐to‐epithelial transition (MET) are strongly induced. The reprogrammable cells will then enter a maturation phase, in which cells undergo a stochastic activation of pluripotency markers. In the late phase, the reprogrammable cells eventually activate the core pluripotency circuitry, complete epigenetic resetting and stabilize into the pluripotent state.^3^, ^4^ Chromatin is dynamically remodelled during cell reprogramming, in which chromatin states shift from open to closed and then again from closed to open.^5^ Chromatin in pluripotent stem cells and iPSCs is less condensed and the ratio between heterochromatin and euchromatin is lower than that in differentiating cells. Reacquiring and maintaining a globally open chromatin state that is accessible for transcriptional activation during reprogramming is very important to the success of cell reprogramming.^6^ However, the mechanisms underlying chromatin remodelling of cell reprogramming are very complex and still need further investigation.

The process of reprogramming somatic cells into induced pluripotent stem cells needs global epigenetic remodelling.^1^ Epigenetic modification has been proven to play key roles during cell reprogramming.^7^ Short hairpin RNAs (shRNAs) screening showed that some chromatin‐modifying enzymes could inhibit or facilitate the reprogramming.^8^ And H3K9 methylation has been identified as a barrier of reprogramming, its removal results in fully reprogrammed iPSCs.^9^ Knockdown of histone deacetylase 2 (HDAC2) promotes the maturation of iPSCs by facilitating TET1 binding and DNA demethylation at the promoters of iPSCs maturation‐related genes.^10^ Ubiquitination is capable of conjugating to a variety of substrates by ubiquitin, including monoubiquitination and polyubiquitination. Monoubiquitination mainly contributes to gene expression and receptor endocytosis. Polyubiquitin chains linked through different lysine residues are involved in protein degradation and signal transduction.^11^, ^12^ Despite recent progress of the functional role of histone modifications and chromatin remodellers, little is known about the role of histone H2B lysine (K) 120 monoubiquitination (H2Bub) in cell reprogramming. As one of the important post‐translational modifications, H2Bub plays vital roles in differentiation,^13^, ^14^ transcription initiation and elongation,^15^, ^16^ meiotic recombination^17^ and other fundamental physiological processes. Fierz B et al found that H2Bub impairs fibre folding and leads to an open and biochemically accessible chromatin conformation.^18^ Two RING finger proteins RNF20 and RNF40 have been identified as the E3 ligase for H2Bub in mammalian cells.^19^ Our previous studies showed that RNF20‐mediated H2Bub regulates meiotic recombination by promoting chromatin relaxation.^17^ Early study found that knockdown of RNF40 increases reprogramming efficiency during human cellular reprogramming,^20^ while recent studies revealed that RNF40 is required for mouse somatic cell reprogramming.^21^ The mechanisms underlying H2Bub mediated somatic cell reprogramming remained unclear. These studies make us think about whether H2Bub takes part in cell reprogramming by promoting chromatin remodelling.

Here, we show that RNF20‐mediated H2Bub is essential for the induction of somatic cell reprogramming by promoting chromatin relaxation. Western blots showed that RNF20 and H2Bub are highly expressed in the early stage of cell reprogramming. We generated iPSCs from *T‐Cre‐Rnf20^Flox/Flox^* MEF cells by infecting viral OSKM vectors. Once *Rnf20* was knocked out, somatic cells could not be reprogrammed to induced pluripotent stem cells. RNA‐seq showed that *Rnf20* knockout mainly affects the early stage of cell reprogramming by impairing the transcription of MET‐related genes and early pluripotency genes. Immunofluorescence and MNase assay revealed that *Rnf20* knockout affects chromatin relaxation and prevents the recruitment of SOX2 to the proper positions on the chromatin, which was further confirmed by ChIP‐seq. Taken together, our findings demonstrate that RNF20‐mediated H2B ubiquitination regulates cell reprogramming by promoting chromatin relaxation, which provides important novel mechanistic insights into the chromatin remodelling of reprogrammable cells.

## 2 MATERIALS AND METHODS

### 2.1 Mice

The *Rnf20^Flox/Flox^* mouse strain was constructed as described previously.^17^ *Rnf20^Flox/Flox^*; *Tamoxifen‐cre* mice were bred from *Rnf20^Flox/Flox^* mice and *Tamoxifen‐cre* mice^22^ were purchased from Jackson laboratory (004682). *Tamoxifen‐cre* is a ubiquitously expressed and tamoxifen‐inducible Cre recombinase. The conditional RNF20 knockout in *T‐Rnf20^Flox/Flox^* MEF cells was obtained by culturing the cells in the presence of 4‐OH tamoxifen during 3 continuous days. All animal experiments were approved by the Animal Research Panel of the Committee on Research Practice of the University of Chinese Academy of Sciences.

### 2.2 Cell culture

MEF cells used for the generation of iPS cells were cultured with DMEM (Invitrogen, 10569‐010) plus 10% FBS (Invitrogen, 10099141) before retroviral infection. For iPS derivation, DMEM/F12 (Invitrogen, 11330‐032) and 20% knockout serum (Invitrogen, 10828‐028) was used instead of DMEM with 15% FBS. Plat‐E cells were cultured with DMEM plus 10% FBS, 1%penicillin–streptomycin (100×) (Invitrogen, 15140‐122), 0.001 mg/ml puromycin (Merck, 540222) and 0.01 mg/ml blasticidins hydrochloride (Invitrogen, R210‐01). iPS cells were cultured on mitomycin‐C (Sigma, M0503) treated MEF cells with DMEM plus 15% FBS, 1000  U ml‐1 LIF (Millipore, ESG1107), 2 mmol/L glutamine (Sigma, G8540), 1 mmol/L sodium pyruvate (Sigma, L7900), 0.1 mmol/L β‐mercaptoethanol (Sigma, M7522) and 0.1 mmol/L non‐essential amino acids (Invitrogen, 11140‐050).

### 2.3 Reprogramming experiments

Reprogramming experiments followed the previously published protocol.^23^ The four retroviral vectors (pMXs‐OCT4, SOX2, C‐MYC and KLF4) were introduced into plat‐E cells using lipofectamine 2000 transfection reagent (Thermo Fisher, 11668019) according to manufacturer's recommendations. After overnight transduction, the medium was replaced. Another 24 hours later, the virus‐containing supernatants were collected and filtered through a 0.45 mm cellulose acetate filter (Millipore, SLHV033RS), supplemented with 4 μg/ml polybrene (Millipore, TR‐1003‐G). MEF cells (seeded at 2 × 10^5^ cells per 10 cm culture dish) were incubated with virus‐containing supernatants for 48 hours with medium change at 24 hours before replacement with regular media. Two days after infection, the infected MEF cells were replaced with 2.5 × 10^4^ cells per 35 mm dish on mitomycin‐C‐treated MEF feeder layers.

### 2.4 Generation of *Rnf20^−/−^* MEF and reprogrammable cells

*Rnf20^Flox/Flox^*; *Tamoxifen‐cre* female mice showed plugs and were pregnant on day 12.5. They were killed and the embryos were isolated as previously described.^24^ For the knockout of RNF20 in the MEF and reprogrammable cells, 4‐OH tamoxifen (Sigma‐Aldrich, H7904) was added to the medium at a final concentration of 0.1 mmol/L to induce the gene knockout for at least 3 days. Cells were incubated in 37°C with 5% CO_2_ in air.

### 2.5 Immunoblotting

Cell extracts were prepared in cold RIPA buffer (25 mmol/L Tris‐HCl, pH 7.6, 150 mmol/L NaCl, 1% Nonidet P‐40 (Sigma, 74 385), 1% sodium deoxycholate (Amresco, D0613), 0.1% sodium dodecyl sulphate (Amresco, 0227) and supplemented with 1 mmol/L phenylmethylsulfonyl fluoride (Amresco, M145) and a protein inhibitor cocktail (Roche Diagnostics, 04693132001). After transient sonication, the cell lysate was incubated on ice for 30 minutes. The samples were centrifuged at 12 000 rpm for 15 minutes to pellet the cell debris, and the supernatant was transferred to a new tube for further analysis. The protein lysates were separated by SDS‐PAGE and electro‐transferred onto a nitrocellulose membrane. The membrane was incubated with corresponding primary antibodies. Primary antibodies included anti‐RNF20 (Proteintech, 21625‐1‐AP, 1:1000), anti‐H2Bub (Cell Signaling Technology, 5546s, 1:1000), anti‐GAPDH (Bo Ao Rui Jing,ab1019t, 1:1000), anti‐Actin (Abmart, M20011, 1:1000), anti‐SOX2 (Santa Claus, sc365823X, 1:1000), anti‐H3 (Proteintech, 17168‐1‐AP, 1:1000) and anti‐H3K9me2 (EASYBIO, BE3283, 1:1000). The next day, the proteins on the membrane were hybridized with Alexa Fluor 680‐conjugated goat anti‐mouse or Alexa Fluor 800‐conjugated goat anti‐rabbit secondary antibodies and scanned using the ODYSSEY Sa Infrared Imaging System (LI‐COR biosciences).

### 2.6 Immunofluorescence and AP staining

Samples were fixed with 4% paraformaldehyde (Solarbio, P1110) for 15 minutes and washed in PBS three times (pH 7.4). Then, the cells were treated with 0.5% Triton X‐100 for 10 minutes and washed in PBS three times. After blocking with 5% bovine serum albumin for 60 minutes, the slides were incubated with a primary antibody in 1% BSA containing 0.3% Triton X‐100 at 4°C overnight. Primary antibodies included anti‐H3K4me2 (EASYBIO, BE3275, 1:200), anti‐H3K4me3 (EASYBIO, BE3276, 1:200), The polyclonal antibody to RNF20 for immunofluorescence was generated in previous studies.^17^ The next day, samples were washed three times with PBS, incubated with FITC‐ or TRITC‐conjugated secondary antibody at a dilution of 1:200 for 1 hours at 37°C. Next, the nuclei were stained with 4',6‐diamidino‐2‐phenylindole (DAPI). Goat anti‐rabbit FITC, goat anti‐mouse FITC, goat anti‐rabbit TRITC and goat anti‐mouse TRITC‐conjugated secondary antibodies, which were purchased from Zhong Shan Jin Qiao. Images were collected immediately using an LSM 780 microscope (Zeiss). An alkaline phosphatase staining kit (Beyotime) was used to detect iPSC colonies according to the manufacturer’ s instructions.

### 2.7 RNA isolation, RT–qPCR

Total RNAs were isolated from reprogrammable cells as previously described.^25^ cDNA was synthesized by the PrimeScriptTM RT Reagent Kit (TaKaRa, RR037A). Real‐time PCR was carried out with a Roche Light Cycler^®^ 480II System, and the results were analysed using the LightCycle480 SW 1.5.1. Table S1 showed the primer sequences used in RT‐qPCR.

### 2.8 Micrococcal nuclease (MNase) sensitivity assay

The assay was performed as previously described.^17^ Briefly, *Rnf20^−/−^* and *Rnf20^+/+^* reprogrammable cells were incubated in a hypotonic buffer (10 mmol/L Hepes, pH 7.9, 10 mmol/L KCl, 1.5 mmol/L MgCl_2_, 0.34 mol/L sucrose, 10% glycerol and 1 mmol/L dithiothreitol (DTT)) supplemented with 0.1% Triton X‐100. After washing in hypotonic buffer without Triton X‐100, the cell nuclei were digested with 4 U MNase in 200 μl reaction buffer (15 mmol/L Tris‐HCl, pH 7.4, 60 mmol/L KCl, 0.25 M sucrose, 1 mmol/L CaCl_2_ and 0.5 mmol/L DTT) at 25°C for the indicated period of time. The reaction was terminated by adding an equal volume of 2 × TNESK buffer (20 mmol/L Tris‐HCl, pH 7.4, 0.2 M NaCl, 2 mmol/L EDTA and 2% SDS) with freshly added proteinase K (0.2 mg/ml). The samples were then incubated 2 hours or overnight at 37°C, and genomic DNA was purified and separated by electrophoresis in a 1.2% agarose gel. The intensity of each lane was quantified using GIS 1D software.

### 2.9 Chromatin immunoprecipitation, library construction

ChIP experiments were performed by using a standard ChIP protocol with modifications. Approximately 1 × 10^6^ cells were crosslinked with 1% formaldehyde for 10 minutes at room temperature. Fixation was stopped by adding Glycine at a final concentration of 125 mmol/L and incubated for 15 minutes at room temperature. The cells were collected by centrifugation for 5 minutes at 500 X *g* and washed twice with ice‐cold PBS. Cells were resuspended in cell lysis buffer (5 mmol/L PIPES, pH 8.0, 85 mmol/L KCl, 0.5% NP‐40 and 1 × protease inhibitor cocktail) and incubated for 10 minutes on ice. The cell nuclei were pelleted at 1000 rpm for 10 minutes at 4°C, and the nuclear pellet was resuspended in nuclei lysis buffer (50 mmol/L Tris‐HCl, pH 8.0, 10 mmol/L EDTA, 1% SDS and 1 × protease inhibitor cocktail) and incubated for 10 minutes on ice. Chromatin DNA was sheared to 250‐600 bp by probe sonicator (SONICS, 630‐0421P) in ultrasonic crusher (Z01121620097228, CP 750W). 5% chromatin fragments were saved as input and the remaining were incubated with magnetic beads conjugated with antibody overnight at 4°C. Beads were sequentially washed four times with high‐salt wash buffer (50 mmol/L HEPES, pH 7.9, 500 mmol/L NaCl, 1 mmol/L EDTA, 0.1% SDS, 1% Triton X‐100, 0.1% deoxycholate and 1 × protease inhibitor cocktail), and two times with TE buffer (10 mmol/L Tris‐HCl, pH 8.0 and 1 mmol/L EDTA). The protein‐chromatin complex was eluted with elution buffer (50 mmol/L Tris‐HCl, pH 8.0, 10 mmol/L EDTA, 1% SDS with 1 μl of proteinase K (20 μg/μl)) for 2 hours at 55°C and de‐crosslinked by incubation overnight at 65°C. The precipitated hybrid fragment was cleaned by phenol:chloroform:isoamyl alcohol once, followed by ethanol precipitation. Sequencing libraries were generated with NEBNext^®^ UltraTM II DNA Library Prep Kit for Illumina (E7645S), according to the manufacturer's instructions. 10 ng of ChIP DNA were used for library preparation. NEBNext Multiplex Oligos for Illumina (Set 1, NEB #E7600) were used for PCR amplification of adaptor‐ligated DNA. Libraries were purified with SPRIselect^®^ Reagent Kit (Beckman Coulter, Inc #B23317). Paired‐end 150 bp sequencing was performed on Illumina NovaSeq 6000 System.

### 2.10 ChIP‐seq data analysis

Data from ChIP ‐Seq experiments were mapped to mm10 genome with Bowtie 2 using default settings, with all duplicates removed by Picard tools (http://broadinstitute.github.io/picard). MACS2 was used to identify peaks with default parameters. Diffbind was used to analyse differences between WT and *Rnf20* knockout reprogrammable cells. For visualization, the aligned reads files (Binary Alignment Map [BAM]) were converted to normalized coverage files (bigWig) using bamCoverage from deepTools. Normalization was performed using bamCoverage from deepTools, with read coverage normalized to 1 × sequencing depth with a re‐normalization by shuffled peaks to eliminate the disturbances from abnormally high‐value regions. Snapshots of the data were constructed using the Integrative Genomics Viewer (IGV). Heatmaps were generated with computeMatrix from deepTools. Metaplots were generated with deepTools. We have provided the detailed information of software used in this study in Table S4, and Table S3 contains the datasets of ChIP‐seq.

### 2.11 RNA‐seq library construction and data processing

Total RNA of WT and *Rnf20* knockout reprogrammable cells at day 3 was harvested using Trizol (Invitrogen). RNA‐seq libraries were prepared with AMPure XP beads mRNA Purification Kit (Beckman) and TruSeq Stranded mRNA Library Prep Kit (Illumina). Sequencing was carried out with Illumina HiSeq 2000. Raw paired‐end sequencing reads were aligned to mouse GRCm38/mm10 reference genome by HISAT2 (2.1.0).^26^ FPKMs (fragments per kilobase of transcript per million mapped reads) were calculated using Cufflinks (v2.1.1). The genes with FPKMs>1, fold changes >2 and FDR<0.01 were identified as the differentially expressed genes. GO analyses were performed using DAVID^27^ and WebGestalt.^28^ Table S2 contains the datasets of RNA‐seq.

### 2.12 MTT assay

Cell viability and cell death under reductive stress or oxidative stress conditions were measured using the 3‐(4,5‐dimethyl‐2‐thiazolyl)‐2, 5‐diphenyltetrazoliumbromide (MTT) assay. Different concentrations of reductive or oxidative stress inducers were added for 24 hours, then MTT (Solarbio, 298‐93‐1) was added and the mixture incubated for 2 hours. DMSO was then added to dissolve the formazan. After 10 minutes the OD was measured at 490 nm.

### 2.13 The generation of whole‐chromosome micronuclei

To generate cells with whole‐chromosome MN, MEF cells were treated with 100 ng ml‐1 nocodazole (Sigma, 31430‐18‐9) for 6 hours. Mitotic cells were collected, washed twice with fresh medium containing 10% FBS, and then plated into medium containing 20% FBS to complete cell division. Cells were then synchronized at G0/G1 by serum starvation for 24 hours, then released into the cell cycle with fresh medium containing 10% FBS and 24 hours later treated with 4 μmol/L dihydrocytochalasin B (Sigma, 39156‐67‐7) for 16 hours to inhibit cytokinesis. Micronuclei were analysed under LSM 780 microscope.

### 2.14 Karyotype analysis

MEF cells were cultured in medium supplemented with colchicine (0.02 mg/ml) to block the cell cycle progression for 3 hours. The metaphase cells were dissociated and collected after washing for three times with PBS. All samples were similarly processed for fixation: cells were pelleted and resuspended in a hypotonic solution of 0.075 M KCL for 20 minutes. Then, the cells were fixed 4 times with 5 ml ice‐cold fixative solution (methanol: acetic acid = 3:1) and collected through centrifugation. Cell pellets were resuspended with 200 μl fixative solution, spread onto the ice‐cold slides for staining. The number and appearance of the chromosomes were analysed under a light microscope after Giemsa staining.

### 2.15 Statistical analyses and reproducibility

Statistical analyses were conducted using GraphPad PRISM version 6.01. All data are presented as the mean ± SEM. The statistical significance of the differences between the mean values for the various genotypes was measured using a Student's *t*‐tests with a paired, 2‐tailed distribution. The data were considered significant when the *P*‐value was less than 0.05 (*), 0.01 (**) or 0.001(***).

## 3 RESULTS

### 3.1 RNF20 is required for cell reprogramming

**FIGURE 1:**
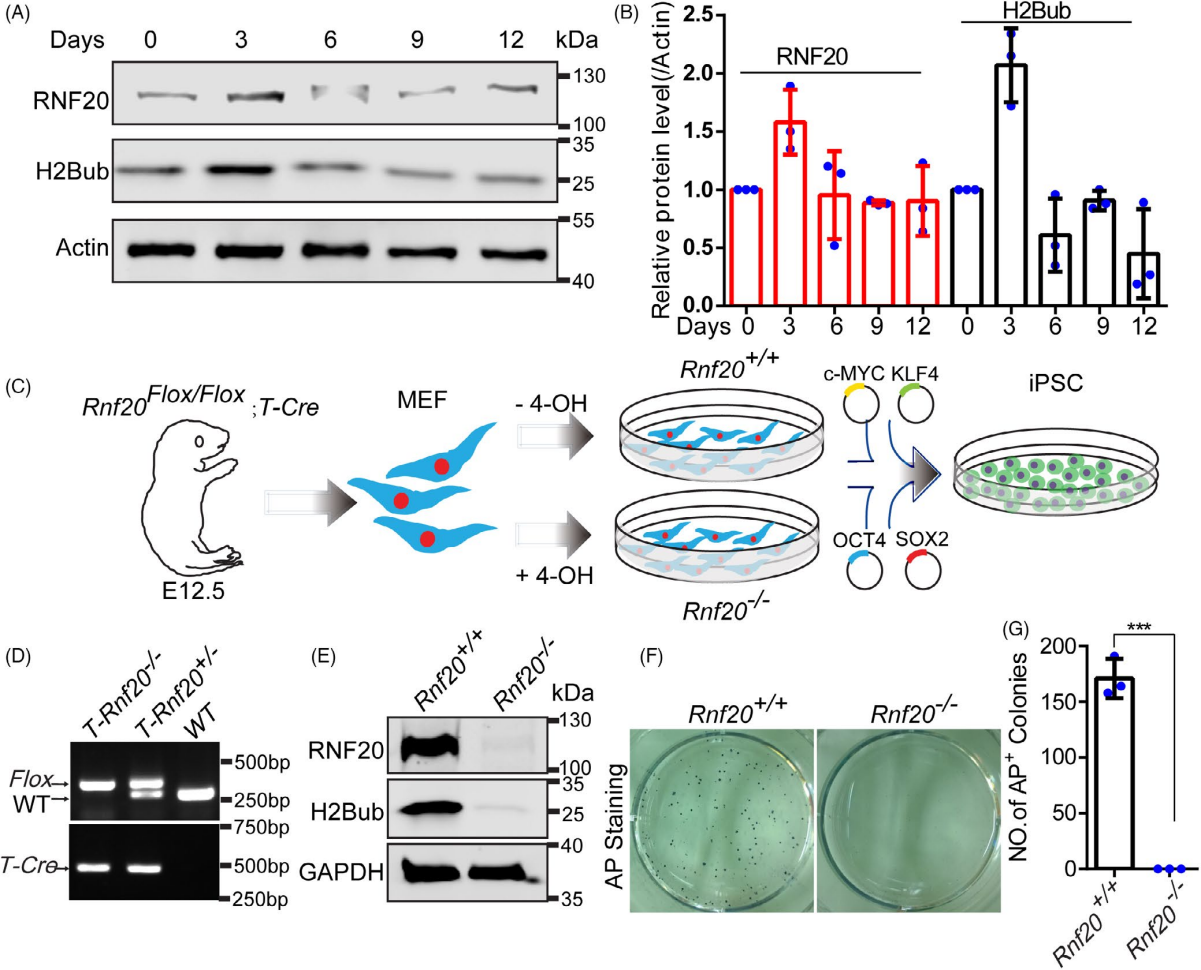
*Rnf20* knockout results in the failure of somatic cell reprogramming. Western blot analysis of the expression pattern of RNF20 and H2Bub during cell reprogramming. Actin served as the loading control. Relative protein levels of RNF20 and H2Bub. (n = 3 independent experiments). Data are presented as mean ± SEM. Schematic depicting the establishment and reprogramming of *Rnf20* knockout MEF cell lines. Representative genotypes of the MEF cell lines. The RNF20 and H2Bub protein levels were reduced in the *T‐Rnf20^−/−^* MEF cell lines by adding 4‐OH tamoxifen for 3 continuous‐days post infection. GAPDH served as the loading control. (F and G) Representative images of AP staining and quantification of AP‐positive colonies in the WT and *Rnf20* knockout reprogrammable cells at day 15. (n = 3 independent experiments). Data are presented as mean ± SEM. ***P < .001

To assess histone H2B lysine (K) 120 monoubiquitination (hereafter referred to as H2Bub) levels at different stages of cell reprogramming, we collected cells at days 0, 3, 6, 9 and 12 from MEF cells, as described by the Yamanaka group, with viral vectors expressing pMXs‐OCT4, SOX2, KLF4 and C‐MYC. Western blots showed that both H2Bub and its E3 ligase, RNF20, are expressed at all stages of reprogramming, with expression levels peaking at day 3 of reprogramming (Figure 1A). Relative protein level analysis also showed that expression of RNF20 and H2Bub peaked at day 3 of cell reprogramming (Figure 1B). Paralleling the occurrence of three reprogramming phases, reprogramming of somatic cells to iPSCs showed two major waves of gene transcriptional activation during the first 3 days and last 3 days of reprogramming, which were separated by a period of relative quiescence.^3^ The first transcriptional wave ensures activation of proliferation metabolism, cytoskeletal organization and cell remodelling response.^3^, ^29^ The expression pattern of RNF20 and H2Bub may be related to the first gene transcriptional wave during cell reprogramming.

We have generated a *Rnf20^Flox/Flox^* mouse model in which *LoxP* was sequentially inserted into the introns flanking exons 2‐4 of the *Rnf20* gene.^17^ To further investigate the functional role of H2Bub in cell reprogramming, *Rnf20^Flox/Flox^* mice were mated with *T‐Cre* (*Tamoxifen‐cre*) mice to produce *Rnf20^Flox/Flox^*; *T‐Cre* mice (Figure 1D). We then selectively deleted RNF20 in *T‐Rnf20^Flox/Flox^* MEF cells by culturing the cells in the presence of 4‐OH tamoxifen during 3 continuous days pre infection (hereafter referred to as *Rnf20* ^−^^/^^−^). iPSCs were then induced from MEF cells carrying either the *Rnf20^+/+^* or the *Rnf20* ^−^^/^^−^ gene and infected with viral OSKM vectors (Figure 1C). Western blot results confirmed that RNF20 was depleted and the expression of H2Bub was dramatically decreased in *Rnf20* ^−^^/^^−^ MEF cells compared with that of *Rnf20^+/+^* MEF cells (Figure 1E). Alkaline phosphatase (AP) staining showed that no AP^+^ colonies were found in *Rnf20* knockout MEF cells at day 15 (Figure 1F,G). These results confirm that *Rnf20* knockout results in the complete failure of somatic cells reprogramming and that is consistent with the effects of *Rnf40* knockout on reprogramming.^21^

### 3.2 RNF20‐mediated H2Bub is essential for the early stage of cell reprogramming

**FIGURE 2:**
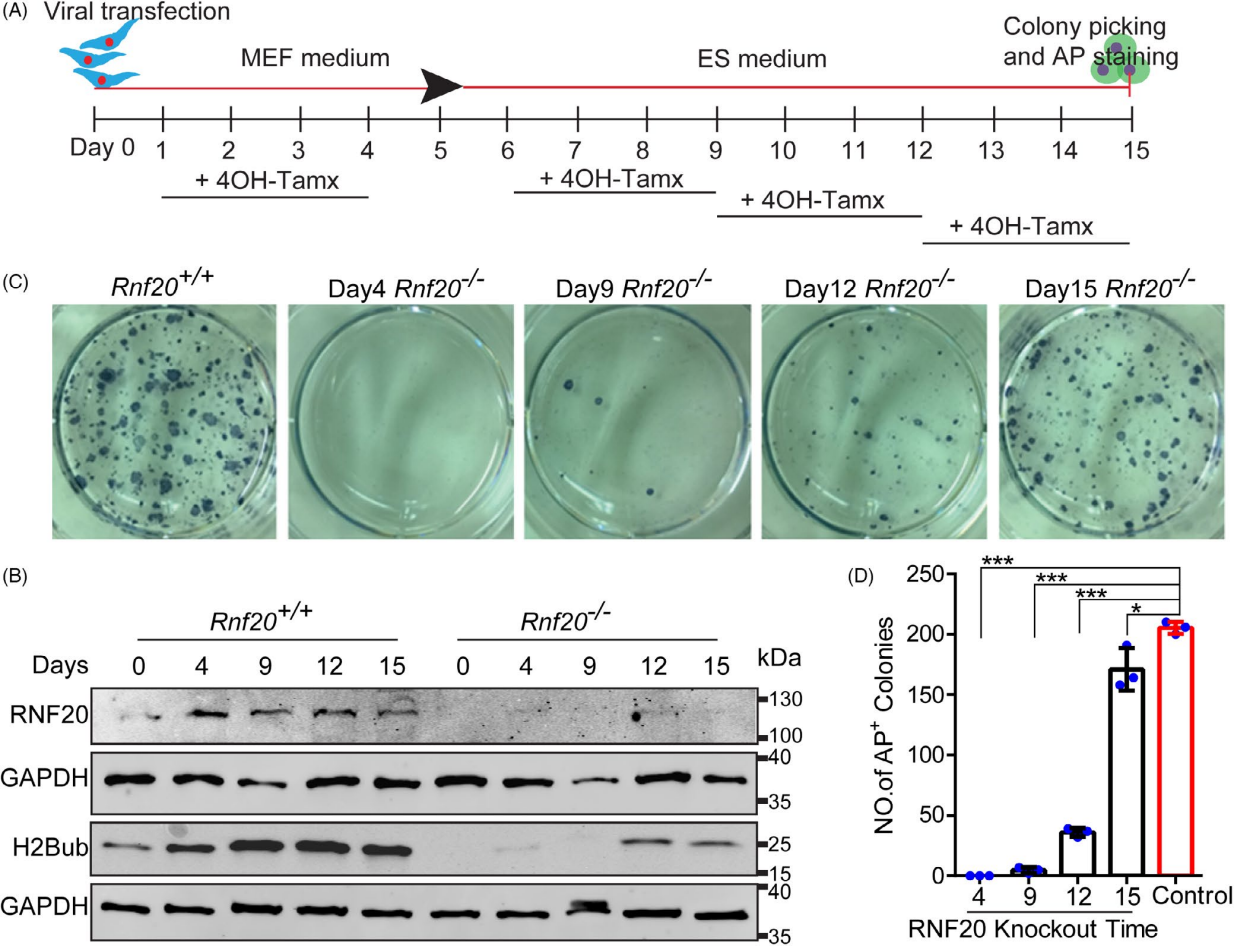
*Rnf20* knockout mainly affects the early stage of cell reprogramming Schematic depicting the treatment window of 4‐OH tamoxifen for *Rnf20* knockout during cell reprogramming. The RNF20 and H2Bub protein levels were reduced in the *Rnf20* knockout reprogramming cells at different time points. GAPDH served as the loading control. Representative images of AP staining in the WT and *Rnf20* knockout reprogrammable cells at different time points. Quantification of AP‐positive colonies in the WT and *Rnf20* knockout reprogrammable cells at different time points. (n = 3 independent experiments). Data are presented as mean ± SEM. *P < .05 and ***P < .001

To explore the duration of H2Bub expression needed for cell reprogramming, we knocked out *Rnf20* at day 4, 9, 12 and 15 by adding 4‐OH tamoxifen (Figure 2A). As shown in Figure 2B, the protein levels of RNF20 and H2Bub were dramatically reduced in *Rnf20* ^−^^/^^−^ reprogrammable cells at every time point compared with those of *Rnf20^+/+^* reprogrammable cells, suggesting that RNF20 and H2Bub were efficiently removed in *Rnf20* ^−^^/^^−^ reprogrammable cells. When we knocked out *Rnf20* at day 4 of cell reprogramming, no AP^+^ colonies were detected. This result is similar to that of *Rnf20* knockout at day 0. AP staining also showed that *Rnf20* knockout at day 9 was relatively modest in impairing cell reprogramming, while *Rnf20* knockout at day 12 and 15 had little effect on cell reprogramming (Figure 2C,D). These results indicate that RNF20 is important for cell reprogramming and mainly required at earlier stages of cell reprogramming (day 0 and day 4). This finding fits well with the observation that RNF20 and H2Bub are highly expressed at the early stage of cell reprogramming (Figure 1A). Previous studies reported that the early transcriptional dynamics are largely dependent on accessible chromatin during cell reprogramming.^30^ All these results suggest that H2Bub mainly participates in the early stage of cell reprogramming.

### 3.3 RNF20 mainly regulates the transcription of MET‐ and early pluripotency‐related genes

**FIGURE 3:**
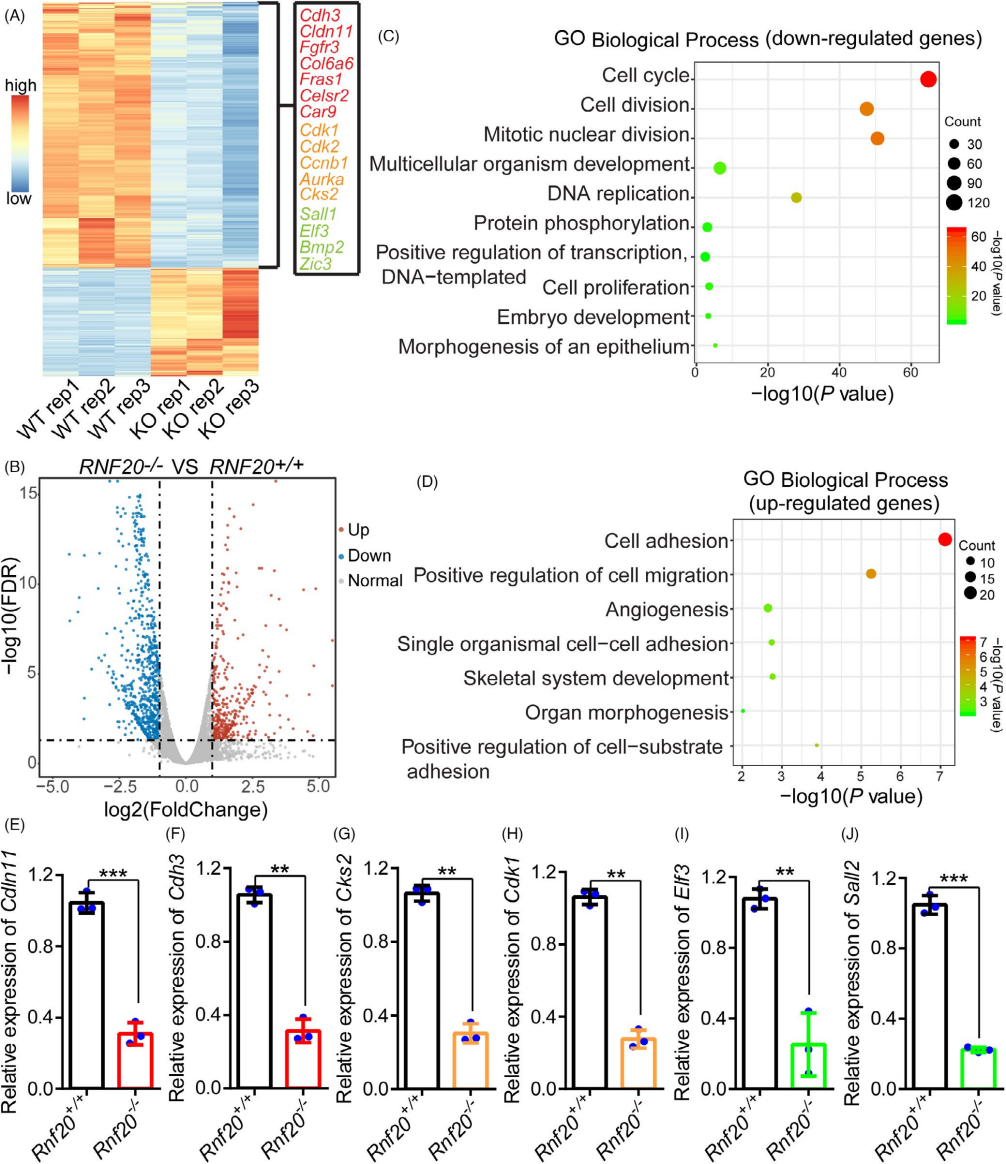
Transcriptomic analysis of *Rnf20* knockout during cell reprogramming. Heatmaps showing significantly upregulated and downregulated genes on day 3 in response to *Rnf20* knockout. Purple representing MET‐related genes, orange representing cell cycle‐related genes, and green representing early pluripotency genes. Volcano plot showing the numbers of upregulated and downregulated genes in *Rnf20* knockout reprogrammable cells were 243 and 594, respectively. (C and D) GO‐Biological process enrichment of downregulated and upregulated genes in *Rnf20* knockout reprogrammable cells versus WT group. (E‐J) RT‐qPCR of *Cdln11, Cdh3, Cks2*, *Cdk1*, *Elf3* and *Sall1* relative to *Gapdh* at day 3 during the reprogramming time course. (n = 3 independent experiments). Data are presented as mean ± SEM. **P < .01 and ***P < .001

Previous studies have demonstrated that RNF20‐mediated H2Bub could regulate gene transcription and its depletion might affect cell growth and genome stability.^31^, ^32^, ^33^ To further investigate the functions of RNF20 at the early phase of cell reprogramming, we performed RNA sequencing (RNA‐seq) at day 3 of OSKM reprogramming in *Rnf20* knockout cells (Figure 3A,B). By comparing the transcriptome of *Rnf20* ^−^^/^^−^ cells and *Rnf20^+/+^* cells, we found that 594 genes were downregulated and 243 genes were upregulated (Figure 3B). The expression of MET‐related genes, cell cycle‐related genes, and pluripotency genes, such as *Cdh3*, *Cldn11*, *Cdk1,* *Aurkb, Sall1* and *Elf3* were obviously^3^, ^34^ downregulated in *Rnf20* ^−^^/^^−^ reprogrammable cells (Figure 3A). Further analysis revealed that epithelial genes failed to be upregulated and genes related to the epithelial‐to‐mesenchymal transition (EMT) were unaffected in *Rnf20* knockout cells (Figure S1A). Importantly, Gene ontology (GO) analysis of different expression genes (DEG) between *Rnf20^‐/‐^* cells and *Rnf20^+/+^* cells revealed that the downregulated genes were strongly linked to cell cycle, DNA replication, cell proliferation, cell division and morphogenesis of epithelium, whereas the upregulated genes were linked to cell adhesion and cell migration processes (Figure 3C,D). The downregulated genes were mainly distributed in the nucleus and involved in protein and DNA binding, whereas the upregulated genes were mainly distributed in the membrane and cytoplasm and associated with calcium ion binding and heparin binding (Figure S1B‐E). These results were further validated by RT‐qPCR (Figure 3E‐3J, Figure S1F,H). Therefore, *Rnf20* knockout leads to an inability to undergo the MET process and a failure to activate the expression of cell cycle‐ and early pluripotency‐related genes.

Considering the results of GO analysis of the downregulated genes involving in cell proliferation, DNA replication and other basic cellular processes. We performed MTT assay to test cell viability and found that the viability of *Rnf20* knockout cells was significantly lower than the WT cells on the fifth day of cell growth (Figure S2A). However, no difference was found in viability between WT and *Rnf20* knockout MEF cells in the early days (1‐4 days) (Figure S2A). Micronuclei (MN) are small, extranuclear chromatin bodies surrounded by a nuclear envelope. Micronuclei have generally been considered as a consequence of DNA damage.^35^, ^36^, ^37^ As shown in Figure S2B,C, *Rnf20* knockout results in increased frequency of micronuclei, suggesting the function of the RNF20 is essential for maintenance of DNA integrity. It is obvious that the *Rnf20* knockout leads to the appearance of broken chromosomes in 10% compared to 2% in WT cells (Figure S2D,E), suggesting that *Rnf20* knockout could cause double‐stranded DNA breaks. Though these results fit well with the previous studies that deficiency of the ubiquitin ligase RNF20 leads to replication stress and chromosomal instability.^38^ In the present study, we mainly focused on the functional role of RNF20 in the early stage of cell reprogramming.

The previously identified ‘first‐wave’ genes are known to be involved in the activation of DNA replication or cell division, and the inhibition of genes associated with cell adhesion and cell‐cell contacts.^3^ These results suggest that the knockout of *Rnf20* leads to the disordered expression of the first transcriptional wave at the early stages of cell reprogramming and the failure to maintain global gene expression.

### 3.4 *Rnf20* knockout affects the recruitment of TFs to their targeted genes

**FIGURE 4:**
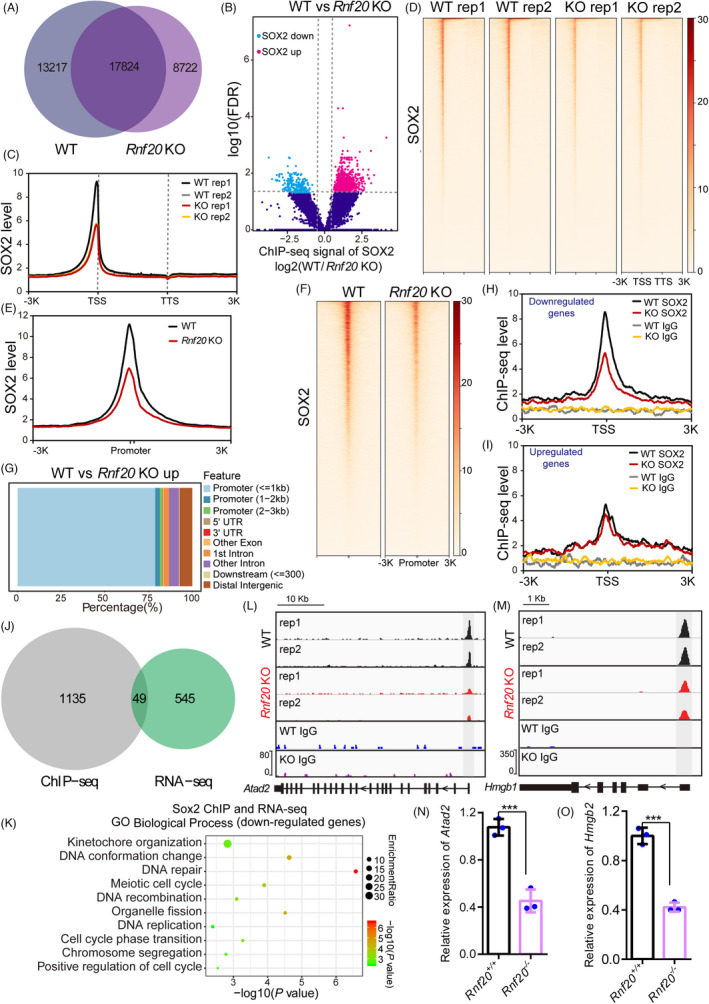
*Rnf20* knockout affects the recruitment of SOX2 to its targeted genes. Venn diagrams showing the overlap of SOX2 ChIP‐seq peaks between the WT and *Rnf20* knockout reprogrammable cells. Volcano plot showing the differences of SOX2 ChIP‐seq signals between the WT and *Rnf20* knockout reprogrammable cells. (C and E) Metaplots showing the ChIP‐seq signals of SOX2 in WT (black line) and *Rnf20* knockout reprogrammable cells (red line). (D and F) The heatmaps of sequence read density for SOX2 in WT and *Rnf20* knockout reprogrammable cells. Distribution of downregulated SOX2 ChIP‐seq peaks in WT and *Rnf20* knockout reprogrammable cells. (H and I) Metaplots of signal intensities for SOX2 in downregulated and upregulated genes sites. Black line representing WT group and red line representing *Rnf20* knockout reprogrammable cells. (J) Venn diagrams showing the overlap of downregulated genes and downregulated SOX2 ChIP‐seq peaks. (K) GO‐Biological process analysis of ChIP‐seq and RNA‐seq for downregulated genes in *Rnf20* knockout reprogrammable cells versus WT reprogrammable cells. (L‐M) Genome views of the level of SOX2 at *Atad2* and *Hmgb1* in WT and *Rnf20* knockout reprogrammable cells. (N‐O) RT‐qPCR showing the expression level of *Atad2* and *Hmgb1* in WT and *Rnf20* knockout reprogrammable cells. *Gapdh* was used as an internal control. (n = 3 independent experiments). Data are presented as mean ± SEM. **P < .01 and ***P < .001

Because OSKM are the major TFs of cell reprogramming and play a key role in gene expression regulation, we further investigated whether *Rnf20* knockout affects the recruitment of OSKM to their target gene sites. To validate this possibility, we performed chromatin immunoprecipitation sequencing (ChIP‐seq) experiments on *Rnf20^+/+^* and *Rnf20* ^−^^/^^−^ reprogrammable cells with SOX2‐specific antibodies. Western blot showed that the expression of SOX2 is unchanged in *Rnf20* ^−^^/^^−^ reprogrammable cells compared with that of *Rnf20^+/+^* reprogrammable cells (Figure S3A). We first analysed previously published SOX2 ChIP‐seq data^39^ and found that there was good overlap between sites bound by SOX2 in our ChIP‐seq results and that of in Zviran et al ChIP‐seq results (Figure S3C), suggesting that our work is consistent with previously published SOX2 ChIP‐seq data. We then compared the SOX2 binding profiles between *Rnf20^+/+^* and *Rnf20* ^−^^/^^−^ reprogrammable cells and found that 17824 SOX2 bound loci were shared by WT and *Rnf20* knockout reprogrammable cells. Other than this, WT reprogrammable cells had unique 13 217 SOX2 bound loci, while *Rnf20* knockout reprogrammable cells had unique 8722 SOX2 bound loci, suggesting that nuclear accumulation of SOX2 is decreased due to *Rnf20* knockout (Figure 4A). DNA sequence motif analysis at the Sox2 ChIP‐seq peaks indicated that there are no differences of the binding motifs between the WT group and *Rnf20* knockout reprogrammable cells (Figure S3B). Further examination of ChIP‐seq signals of all SOX2 bound loci revealed that a large number of SOX2 bound loci decreased abruptly (Figure 4B). Within unique and common SOX2 bound loci in WT and *Rnf20* knockout reprogrammable cells, SOX2 preferentially bound to upstream regions of transcription start sites (TSSs) and the enhancer regions (Figure 4C‐4D, Figure S3D,E). Further analysis showed that *Rnf20* knockout has no effects on the recruitment of SOX2 to the enhancer regions, while the enrichment at promoter regions clearly decreased due to RNF20 depletion (Figure 4E‐4F and Figure S3F), which is consistent with lacking H2Bub1 enrichment at the enhancer regions.^40^, ^41^ These results suggest that RNF20‐mediated H2Bub mainly regulate the recruitment of SOX2 or other transcription factors to the promoter regions, but not the enhancer regions.

Further examination of downregulated SOX2 bound loci confirmed that SOX2 were preferentially enriched at promoter regions (Figure 4G,4H), while no significant ChIP‐seq signal changes were observed between WT and *Rnf20* knockout reprogrammable cells in upregulated genes (Figure 4I). These results suggest that *Rnf20* knockout affects the recruitment of SOX2 to the promoter regions of downregulated genes. We next compared downregulated genes with RNA‐seq and downregulated SOX2 signals with ChIP‐seq, finding 594 downregulated genes in *Rnf20* knockout reprogrammable cells when compared with WT reprogrammable cells. Among the 594 genes, 49 genes were also associated with decreased SOX2 signals upon *Rnf20* knockout (Figure 4J). Further analysis revealed that the genes with decreased SOX2 binding and downregulated expression in *Rnf20* knockout reprogrammable cells are involved in kinetochore organization, DNA conformation change and DNA replication^42^, ^43^ (Figure 4K‐4O). These results suggest a positive correlation between SOX2 binding and gene expression, and that the knockout of *Rnf20* might affect the recruitment of reprogramming TFs to their target genes, ultimately impeding the initiation of ‘first‐wave’ gene expression.

### 3.5 RNF20‐mediated H2Bub affects gene transcription by regulating chromatin conformation

**FIGURE 5:**
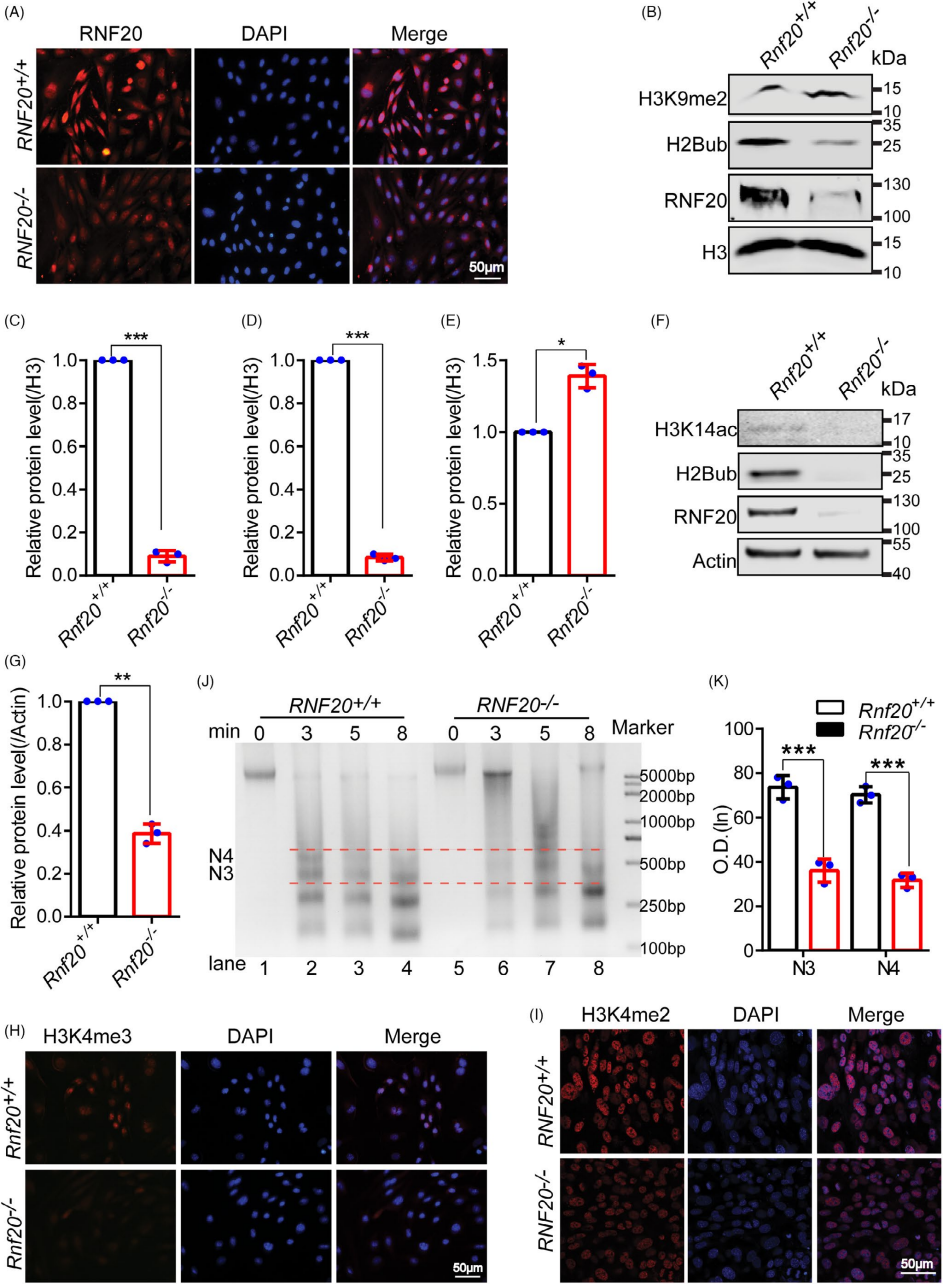
RNF20‐mediated H2Bub affects somatic cell reprogramming by promoting chromatin relaxation. (A) Representative images of RNF20 in the WT and *Rnf20* knockout reprogrammable cells. (B) H3K9me2 protein levels increased when RNF20 and H2Bub protein levels were reduced in *Rnf20* knockout reprogrammable cells. H3 served as the loading control. (C) Relative protein levels of RNF20 in the WT and Rnf20 knockout reprogrammable cells. (n = 3 independent experiments). Data are presented as mean ± SEM. ***P < .001. (D) Relative protein levels of H2Bub in the WT and Rnf20 knockout reprogrammable cells. (n = 3 independent experiments). Data are presented as mean ± SEM. ***P < .001. (E) Relative protein levels of H3K9me2 in the WT and Rnf20 knockout reprogrammable cells. (n = 3 independent experiments). Data are presented as ± SEM. *P < .05. (F) H3K14ac protein levels decreased when RNF20 and H2Bub protein levels were reduced in *Rnf20* knockout reprogrammable cells. Actin served as the loading control. (G) Relative protein levels of H3K14ac in the WT and *Rnf20* knockout reprogrammable cells. (n = 3 independent experiments). Data are presented as mean ± SEM. **P < .01. (H and I) Representative images of H3K4me3 and H3K4me2 in the WT and *Rnf20* knockout reprogrammable cells. (J) Micrococcal nuclease (MNase) sensitivity assays were used to detect the chromatin compaction status in isolated WT and *Rnf20* knockout reprogrammable cells. (K) Quantification of the N3 and N4 intensities of lanes 2 and 6 in (D) using GIS 1D software; N3 and N4 indicate the number of nucleosomes. (n = 3 independent experiments) Data are presented as mean ± SEM. ***P < .001

Recent findings indicate that an open chromatin state is accessible for transcriptional activation and protein‐protein interactions in pluripotency and reprogrammable cells.^6^ It has been reported that H2Bub is involved in regulating chromatin conformation.^18^ Based on the effects of RNF20 and H2Bub on gene expression and cell reprogramming, we reasoned that RNF20 may affect gene transcription through regulating chromatin structure and the recruitment of TFs to the proper locations within the chromatin. H3K9 dimethylation (H3K9me2) is a repressive transcription histone marker. After confirming that RNF20 was depleted and the expression of H2Bub was dramatically decreased in *Rnf20* ^−^^/^^−^ reprogrammable cells compared with that of *Rnf20^+/+^* reprogrammable cells (Figure 5A,B,D,E), we found that H3K9me2 was significantly increased in *Rnf20* ^−^^/^^−^ reprogrammable cells compared with that of *Rnf20^+/+^* reprogrammable cells (Figure 5B,C). Moreover, The acetylation status of H3K14 (H3K14ac), which is an active transcription histone marker and directly involved in regulating chromatin relaxation.^44^, ^45^ The total level of H3K14ac was reduced dramatically in the *Rnf20* knockout reprogrammable cells (Figure 5F,G). H3K4 trimethylation (H3K4me3), H3K4 dimethylation (H3K4me2) and H3K9 dimethylation (H3K9me2) are also active transcription histone markers. We found that H3K4me3 and H3K4me2 levels were reduced dramatically in *Rnf20* ^−^^/^^−^ reprogrammable cells (Figure 5H,I), these results suggest that RNF20 might affect gene transcription through regulating chromatin structure. To further confirm the changes in chromatin structure of *Rnf20* knockout reprogrammable cells, we performed micrococcal nuclease (MNase) sensitivity assays. We found that *Rnf20* ^−^^/^^−^ chromosomes were more resistant to MNase digestion than *Rnf20^+/+^* chromosomes (lanes 2 and 6, lanes 3 and 7) (Figure 5J,K), suggesting that RNF20‐mediated H2B ubiquitination promotes chromatin relaxation during cell reprogramming. Together, these results suggest that RNF20‐mediated H2B ubiquitination relaxes the chromatin structure during the early phase of cell reprogramming, allowing the recruitment of TFs to the proper chromatin location. Therefore, the altering epigenetic environment cooperates with TFs to regulate chromatin remodelling.

## 4 DISCUSSION

Histone modifications play a significant role in somatic cell reprogramming by the regulation of chromatin configuration and gene expression.^46^, ^47^ RNF20 is the major E3 ubiquitin ligase for H2B ubiquitination and is required for development as targeted disruption of RNF20 leads to preimplantation embryonic lethality in mice.^48^ RNF20‐mediated H2B ubiquitination was reported to be required for embryonic stem cell differentiation, suggesting that RNF20 regulates cell fate decisions like other reprogramming‐related factors.^13^, ^14^, ^49^ In this study, we found that H2B ubiquitination mediated chromatin relaxation is essential for the induction of somatic cell reprogramming.

Chromatin is dynamically remodelled during cell reprogramming.^5^ It was recently reported that widespread chromatin reconfiguration and transcription factor occupancy occurs early during reprogramming.^50^ They found that OCT4/SOX2 occurs primarily in regions with MEF promoters during the early stage of cell reprogramming, which is consistent with our work. Furthermore, OCT4 and SOX2 extensively target the accessible chromatin and recruit somatic transcription factors to transiently accessible sites.^50^ Our results showed reduced SOX2 binding due to the condensed chromatin states in *Rnf20^−/−^* reprogrammable cells (Figures 4B, Figure 5D,E). Therefore, some key transcription factors may be regulated by both Yamanaka factors and RNF20‐mediated H2B ubiquitination. Although we only examine the relationship between SOX2 binding sites and their expression, we believe our conclusions are likely applied to OCT4 and even other Yamanaka factors. There is no doubt that further studies are needed to substantiate this point and further dissect the detailed regulation mechanism.

One study has described the effects of *Rnf40* knockout on reprogramming, they try to explain the function of RNF40‐mediated H2Bub by comparing the occupancy of H3K4me3 and H3K27me3 of MEF cells with that of ESCs.^21^ However, Chromatin is dynamically remodelled during cell reprogramming, we found that RNF20 and H2Bub are highly expressed at the early stage of cell reprogramming. We focused our studies on the early stage of cell reprogramming, and uncover the mechanism underlying the initiation of somatic cell reprogramming. The knockout of *Rnf20* in reprogrammable cells affects chromatin relaxation by reducing H2B ubiquitination and normal levels of epigenetic modification, thus preventing the recruitment of SOX2 and most likely other factors to their proper locations on the chromosome and impairing the transcription of pluripotency genes and MET‐related genes during the early stages of cell reprogramming. Our results not only uncover a previously unknown function of RNF20‐mediated H2B ubiquitination during the early stages of cell reprogramming, but also provide important novel mechanistic insights into the chromatin remodelling of reprogrammable cells.

## 5 ETHICS STATEMENT

Animal experimentation: All of the animal experiments were performed according to approved institutional animal care and use committee (IACUC) protocols (#08‐133) of the Institute of Zoology, Chinese Academy of Sciences. All surgery was performed under sodium pentobarbital anaesthesia, and every effort was made to minimize suffering.

## CONFLICTS OF INTERESTS

Liying Wang, Zhiliang Xu, Libin Wang, Chao Liu, Huafang Wei, Ruidan Zhang, Lina Wang, Wenwen Liu, Sai Xiao, Yinghong Chen, Wei Li and Wei Li declare that they have no conflict of interest.

## AUTHOR CONTRIBUTIONS

Liying Wang and Zhiliang Xu performed the majority of the experiments and analysed the data. Libin Wang helped with the establishment of mouse iPSC cultures. Chao Liu contributed to ChIP experiments and analysed the data. Huafang Wei, Ruidan Zhang, Lina Wang and Wenwen Liu performed some experiments in isolating MEF cells. Sai Xiao and Yinghong Chen analysed the data. Wei Li and Wei Li designed the study and prepared the manuscript.

## Supporting information

Fig S1‐S3

Table S1

Table S2

Table S3

Table S4

## Data Availability

All raw high‐throughput sequencing data have been uploaded to the GEO, and the GEO accession number is GSE165887. The authors declare that all other data supporting the findings of this study are available within the article and its Supplementary Informationiles or from the corresponding author on reasonable request.
