# Supplementary material for: Histone H2B ubiquitination mediated chromatin relaxation is essential for the induction of somatic cell reprogramming
Source: Cell Prolif. 2021 Jun 22;54(8):e13080. doi: 10.1111/cpr.13080 (PMC8349662; doi:10.1111/cpr.13080)
Supplement: Supplementary file 1 — Fig S1‐S3 [file CPR-54-e13080-s002.docx]

**Supplemental Information**

**SUPPLEMENTAL FIGURES**

**
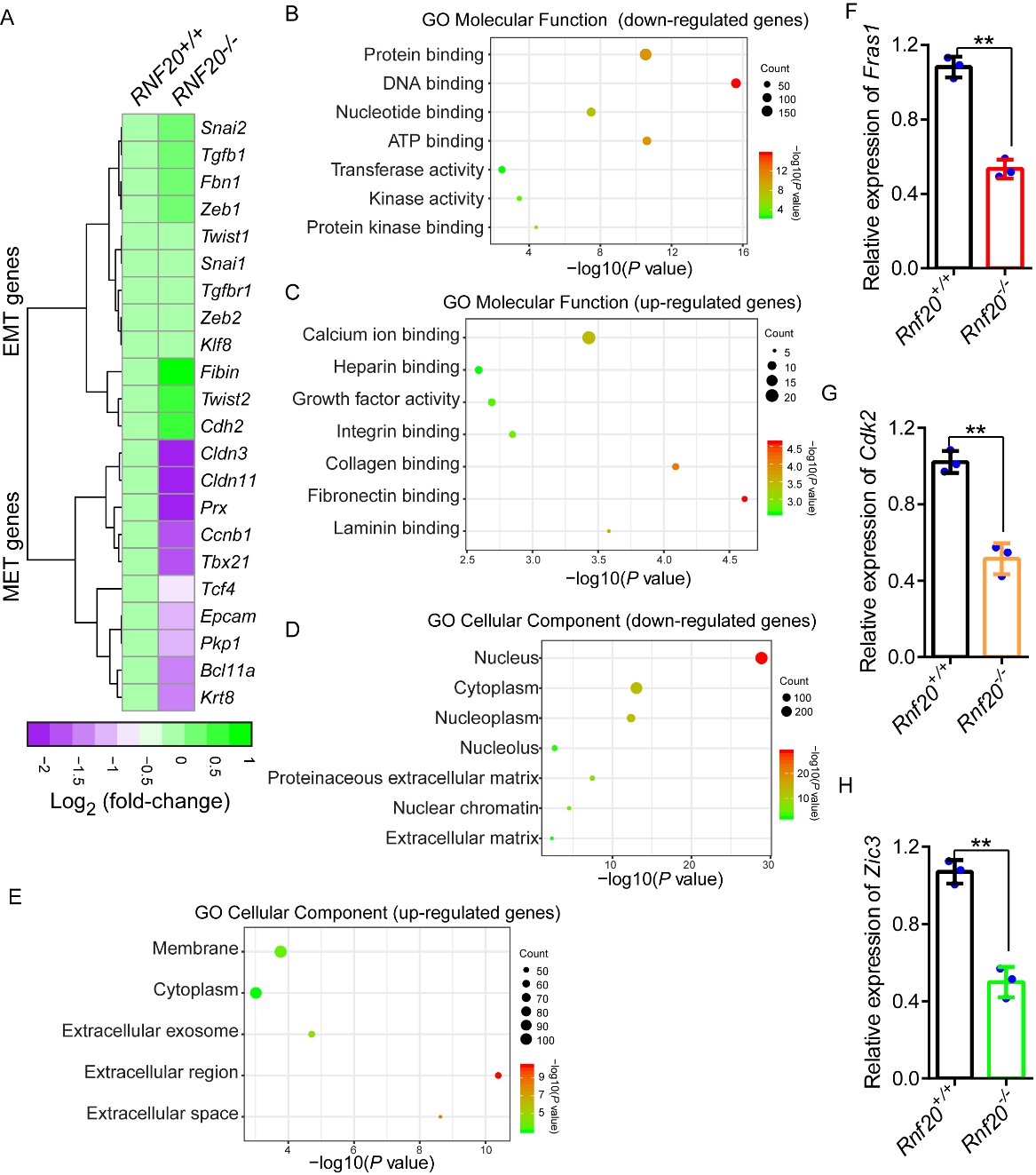
**

**Figure S1. RNA-seq analysis of *Rnf20* knockout during cell reprogramming.**

1. Heatmap showing the fold-change of selected EMT and MET -related genes from the RNA-seq analysis.

(B and C) GO-Molecular function enrichment of downregulated genes and upregulated genes in *Rnf20*-knockout reprogrammable cells versus WT reprogrammable cells.

(D and E) GO-Cellular component enrichment of downregulated genes and upregulated genes in *Rnf20*-knockout reprogrammable cells versus WT reprogrammable cells.

(F-H) RT-qPCR of *Fras1, Cdk2* and *Zic3* relative to *Gapdh* at day 3 during the reprogramming time course. (n = 3 independent experiments). Data are presented as mean ± SEM. **p < 0.01.


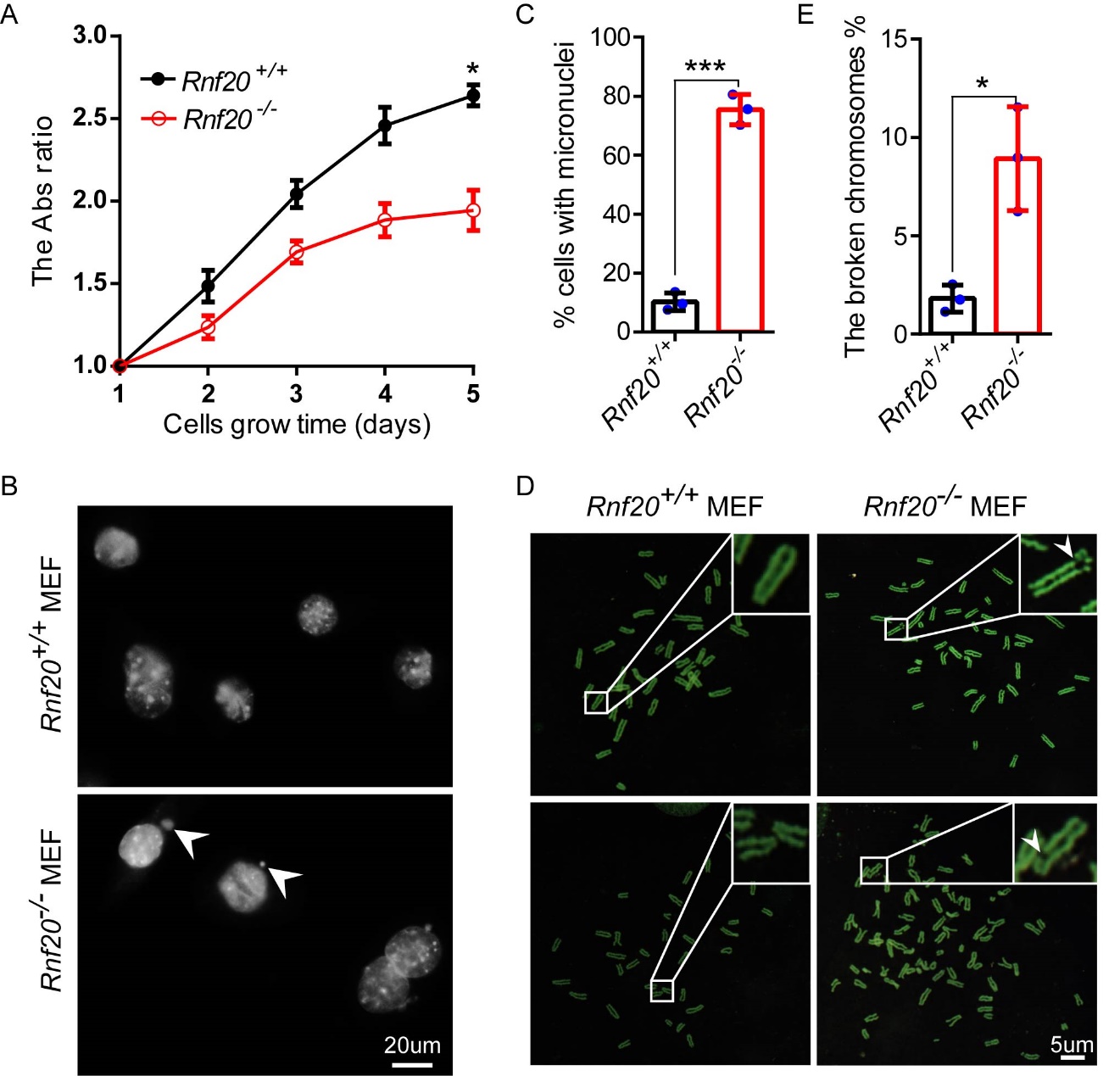


**Figure S2. *Rnf20* knockout results in DNA damage.**

(A) The viability of the cells was monitored in the WT and *Rnf20*-knockout MEF cells by MTT assay. (n = 3 independent experiments). Data are presented as mean ± SEM. *p < 0.05.

(B) Representative images showing micronuclei in MEF cells after *Rnf20-*knockout.

(C) Quantification of cells with micronuclei in the WT and *Rnf20*-knockout MEF cells. (n = 3 independent experiments). Data are presented as mean ± SEM. ***p < 0.001.

(D) Representative pictures of the karyotypes of WT and *Rnf20*-knockout MEF cells.

(E) Quantification of the broken chromosomes in the WT and *Rnf20*-knockout MEF cells. (n = 3 independent experiments). Data are presented as mean ± SEM. *p < 0.05.


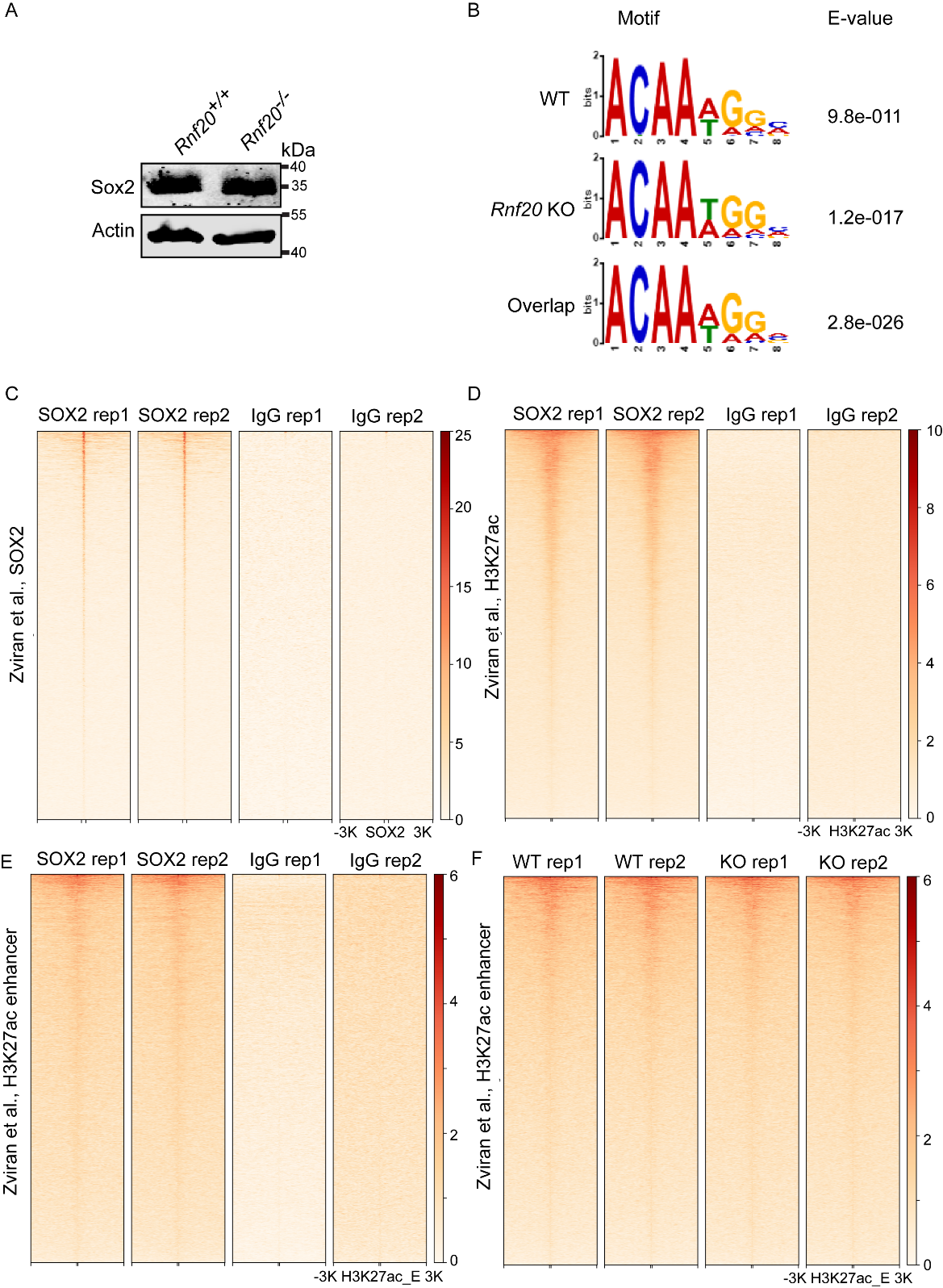


**Figure S3. *Rnf20* knockout has no effects on the recruitment of SOX2 to the enhancer regions.**

(A) The expression of SOX2 in WT and *Rnf20*-knockout reprogrammable cells. Actin served as the loading control.

(B) Motifs identified by MEME in WT and *Rnf20*-knockout reprogrammable cells.

(C) Heatmaps of SOX2 ChIP-seq signal at previously reported SOX2 binding sites.

(D) Heatmaps of SOX2 ChIP-seq signal at H3K27ac binding sites.

(E) Heatmaps of SOX2 ChIP-seq signal at the enhancer region, which previously detected by H3K27ac ChIP-seq.

(F) Heatmaps of SOX2 ChIP-seq signal at the enhancer region in WT and *Rnf20*-knockout reprogrammable cells.
